# Nsp2 replicase-mediated viral uncoating in porcine alveolar macrophages contributes to the attenuation of PRRSV-2 live attenuated vaccine

**DOI:** 10.1128/jvi.00636-25

**Published:** 2025-08-04

**Authors:** Yuan-Zhe Bai, Hu Xu, Yong-Gang Liu, Yue Sun, Shi-Jia Xu, Meng-Xin Wang, Qian Wang, Zhi-Jun Tian, Chao-Liang Leng, Gang Wang, Tong-Qing An, Xue-Hui Cai, Hong-Liang Zhang, Yan-Dong Tang

**Affiliations:** 1 State Key Laboratory for Animal Disease Control and Prevention, Harbin Veterinary Research Institute of Chinese Academy of Agricultural Sciences, Harbin, China; 2 Henan Key Laboratory of Insect Biology in Funiu Mountain, Henan Provincial Engineering and Technology Center of Animal Disease Diagnosis and Integrated Control, Nanyang Normal University 71072, Nanyang, Henan, China; 3 Shandong Agricultural University 34734, Tai'an, Shandong, China; 4 Heilongjiang Provincial Research Center for Veterinary Biomedicine, Chinese Academy of Agricultural Sciences Harbin Veterinary Research Institute 111613 , Harbin, China; 5 Heilongjiang Provincial Key Laboratory of Veterinary Immunology, Chinese Academy of Agricultural Sciences Harbin Veterinary Research Institute 111613 , Harbin, China; Loyola University Chicago - Health Sciences Campus, Maywood, Illinois, USA

**Keywords:** PRRSV, nsp2, live attenuated vaccine, uncoating, primary PAMs

## Abstract

Type 2 porcine reproductive and respiratory syndrome virus (PRRSV-2) poses a major threat to global swine production. While live attenuated vaccines (LAVs) remain the most effective countermeasure, the molecular mechanisms underlying PRRSV-2 attenuation remain enigmatic. Here, we reveal that PRRSV-2 LAVs exhibit impaired replication in their primary cellular targets-porcine alveolar macrophages (PAMs), with viral uncoating defects being the critical replication barrier. Mechanistically, we identified nonstructural protein 2 (nsp2) as the key viral determinant orchestrating this attenuation phenotype. Strikingly, the substitution of the nsp2 from the vaccine strain into a highly pathogenic PRRSV (HP-PRRSV) significantly attenuated virulence in piglets while maintaining immunogenicity. The chimeric virus elicited robust protective immunity against HP-PRRSV challenge. Our findings elucidated that nsp2-mediated viral uncoating contributes to PRRSV-2 LAV attenuation and established a proof-of-concept strategy for rational PRRSV-2 vaccine design.

**IMPORTANCE:**

Live attenuated vaccines (LAVs) are predominantly used for the management of PRRSV infection; however, limited knowledge exists regarding the mechanisms underlying PRRSV attenuation. Enhancing our understanding of the mechanism by which viruses are attenuated would accelerate the development of optimal live attenuated vaccines against PRRSV. In the present study, we discovered that commercial PRRSV LAVs failed to uncoat inside porcine alveolar macrophages, thereby identifying a novel mechanism by which these LAVs achieve attenuation. Notably, we identified nsp2, a virion protein, as a key factor contributing to the attenuation of PRRSV. Furthermore, we demonstrated that the substitution of the nsp2-coding region with its counterpart derived from a commercial LAV enabled the rapid attenuation of highly virulent strains while providing effective protection against subsequent challenges. Our findings elucidated the feasibility of converting virulent PRRSV into an attenuated vaccine candidate in a timely manner.

## INTRODUCTION

Live attenuated vaccines (LAVs) represent the most successful tool for combating viral diseases (1–3). Traditional LAVs involve the adaptation of pathogenic viruses to cell cultures, novel hosts, or suboptimal environments to diminish their virulence while preserving immunogenicity (3). However, the precise mechanisms responsible for this attenuation remain largely unclear for the majority of LAVs. Porcine reproductive and respiratory syndrome viruses (PRRSVs) are important swine pathogens that impose substantial financial burdens on the global swine industry (4). PRRSVs belong to the family *Arteriviridae*, order *Nidovirales*, and are typically categorized into two species: PRRSV-1 and PRRSV-2 (5–7). Clinically, PRRSVs lead to severe respiratory diseases in piglets and reproductive disorders in sows (7). PRRSV mainly targets macrophages and disrupts the immune system, which, in turn, leads to severe secondary bacterial infections (8, 9). China is the largest pig producer and has the largest pork consumption market in the world. PRRSV-2 has been identified as an epidemic strain in China since its outbreak, leading to significant losses for most farms (10–12). The first outbreak of PRRSV in China occurred at the end of 1995, and the CH-1a strain was isolated by Guo et al. (11). In the subsequent decade, PRRSV primarily caused devastating abortions in pregnant sows. Moreover, in 2006, China experienced an outbreak of highly pathogenic PRRSVs (HP-PRRSVs), including representative strains such as HuN4 (13), JXA1 (14), and JXwn06 (11). Subsequent evolutionary analysis revealed that these HP-PRRSVs originated from CH1a-like strains (10). Furthermore, recent outbreaks of NADC30-like and NADC34-like PRRSVs have also been reported in China (15, 16).

In China, commercial live attenuated PRRSV vaccines have been widely used and have shown effectiveness in controlling the virus. Their development primarily involves continuous serial passages of virulent strains on cultured cells, predominantly Marc-145 cells (a monkey kidney cell line) (17). For example, the first commercial PRRSV LAV in China was the CH-1R strain, which was developed through 160 serial passages of the virulent CH-1a strain on Marc-145 cells (17). Similarly, HuN4-F112, JXA1-R, TJM, and GDr180 vaccine strains were generated by passaging their corresponding highly pathogenic strains on Marc-145 cells for 112, 80, 92, and 180 generations, respectively (18). However, these vaccines exhibit robust protection primarily against homologous strains, while demonstrating limited efficacy against heterologous strains such as NADC30-like and NADC34-like PRRSVs (19–21). Consequently, a comprehensive elucidation of the attenuation mechanisms underlying commercial LAVs will facilitate the rapid development of novel vaccines targeting prevalent epidemic strains.

Nonstructural protein 2 (nsp2) is a versatile protein involved in the lifecycle of PRRSV (9, 22–26). PRRSV nsp2 is a multitransmembrane viral protein comprised of five structural domains (27). As the largest viral replicative enzyme, nsp2 facilitates the assembly of viral transcription-replication complexes (28). The N-terminal domain of nsp2 harbors a cysteine protease domain (CP), exhibiting both cis- and trans-cleavage activities (22, 29, 30). The highly variable region of nsp2 governs viral mRNA synthesis (25). Furthermore, nsp2 plays a pivotal role in modulating inflammation and counteracting innate immune responses (23, 31). In addition, nsp2 regulates cellular autophagy and apoptosis processes (32, 33). Different isoforms of PRRSV nsp2 have been identified, including full-length nsp2, nsp2TF, and nsp2N (22). The expression of nsp2TF is mediated by a −2 programmed ribosomal frameshift (PRF) mechanism (34). The expression of nsp2N is mediated by a −1 programmed ribosomal frameshift (PRF), which produces an immediate termination codon, resulting in the production of a truncated form of the nsp2 protein referred to as nsp2N (35). Other cleavage isoforms have also been identified (26). The three reported forms of PRRSV nsp2 (nsp2, nsp2TF, and nsp2N) are essential for suppressing the host immune response (9, 31, 36). In addition, PRRSV nsp2TF interacts with the viral GP5 and M proteins to facilitate PRRSV assembly (37). In our recent study, we demonstrated that full-length nsp2 replicase contributes to viral assembly in highly pathogenic PRRSV-2 (38).

In this study, we initially assessed the replication efficiency of commercially available PRRSV LAVs in porcine alveolar macrophages (PAMs), which are the predominant target cells for PRRSV infection in pigs. Intriguingly, our findings revealed that these PRRSV LAVs exhibited reduced propagation capability in PAMs compared with that of the virulent HuN4 strain. Subsequent investigations demonstrated that the restriction of vaccine strain infection in PAMs occurred after virus attachment and entry but prior to viral RNA synthesis, specifically at the uncoating stage. Notably, our results revealed the involvement of nsp2 in facilitating the uncoating process of PRRSV LAVs within PAMs. Finally, we discovered that substituting nsp2 from a CH-1R vaccine strain attenuated highly pathogenic PRRSV in piglets and that immunization with this chimeric virus conferred effective protection against HP-PRRSV challenge.

## RESULTS

### PRRSV-2 commercial LAVs fail to propagate in PAMs

**Fig 1:**
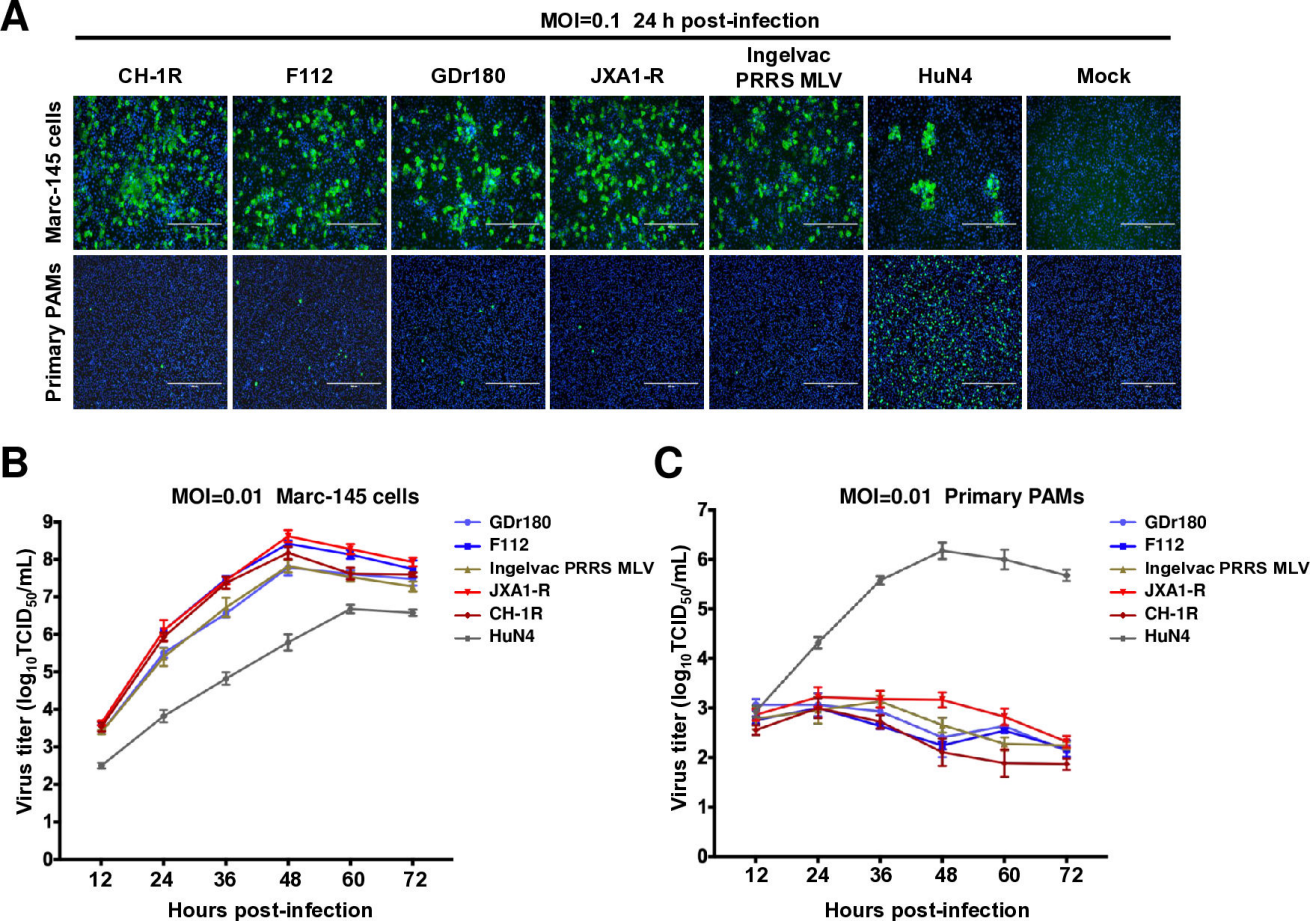
The live attenuated vaccines for PRRSV-2 fail to replicate efficiently in primary PAMs. (**A**) Marc-145 cells and primary PAMs were infected with CH-1R, F112, GDr180, JXA1-R, Ingelvac PRRS MLV, or HuN4 at MOI of 0.1; uninfected cells were used as controls. At 24 hours post-infection, the cells were subjected to an IFA with an antibody against PRRSV N protein. Scale bars, 400 µm. (**B**) Viral replication kinetics in Marc-145 cells and (**C**) primary PAMs. The cells were infected with CH-1R, F112, GDr180, JXA1-R, VR-2332, or HuN4 at MOI of 0.01. At the indicated time points post-infection, the viruses were harvested and titered in Marc-145 cells.

PAMs are the major target of PRRSV in pigs *in vivo*, and PRRSV adaptability in PAMs directly contributes to PRRSV virulence (39). LAVs are developed by adapting Marc-145 cells; therefore, we first evaluated the replication ability of commercial PRRSV LAVs on both Marc-145 cells and primary PAMs. In this study, five LAVs, namely CH-1R, F112, JXA1-R, GDr180, and Ingelvac PRRS MLV (its parental strain VR-2332), were selected. In addition, a highly pathogenic PRRSV HuN4 strain was used as a control. Marc-145 cells and primary PAMs were infected with the indicated viruses at multiplicity of infection (MOI) of 0.1. At 24 hours post-infection, viral replication was assessed via an immunofluorescence assay (IFA). The results demonstrated that all five PRRSV vaccine strains and PRRSV HuN4 replicated efficiently in Marc-145 cells (Fig. 1A). Interestingly, only a limited number of primary PAMs were susceptible to infection by vaccine strains, while the highly virulent PRRSV HuN4 efficiently replicated in PAMs (Fig. 1A). The replication kinetics of these viruses were subsequently evaluated in primary PAMs and Marc-145 cells. Primary PAMs and Marc-145 cells were infected with the indicated virus at MOI of 0.01. The virus was then collected and quantified at indicated time points post-infection. The results demonstrated that all the tested PRRSV vaccine strains and the highly virulent HuN4 strain replicated efficiently in Marc-145 cells (Fig. 1B). However, these vaccine strains exhibited limited infectivity in primary PAMs compared with the efficient replication observed for the highly virulent HuN4 strain (Fig. 1C). These findings suggested that LAVs have lost their infectivity in primary PAMs.

### Live attenuated PRRSV vaccines can attach and enter primary PAMs

**Fig 2:**
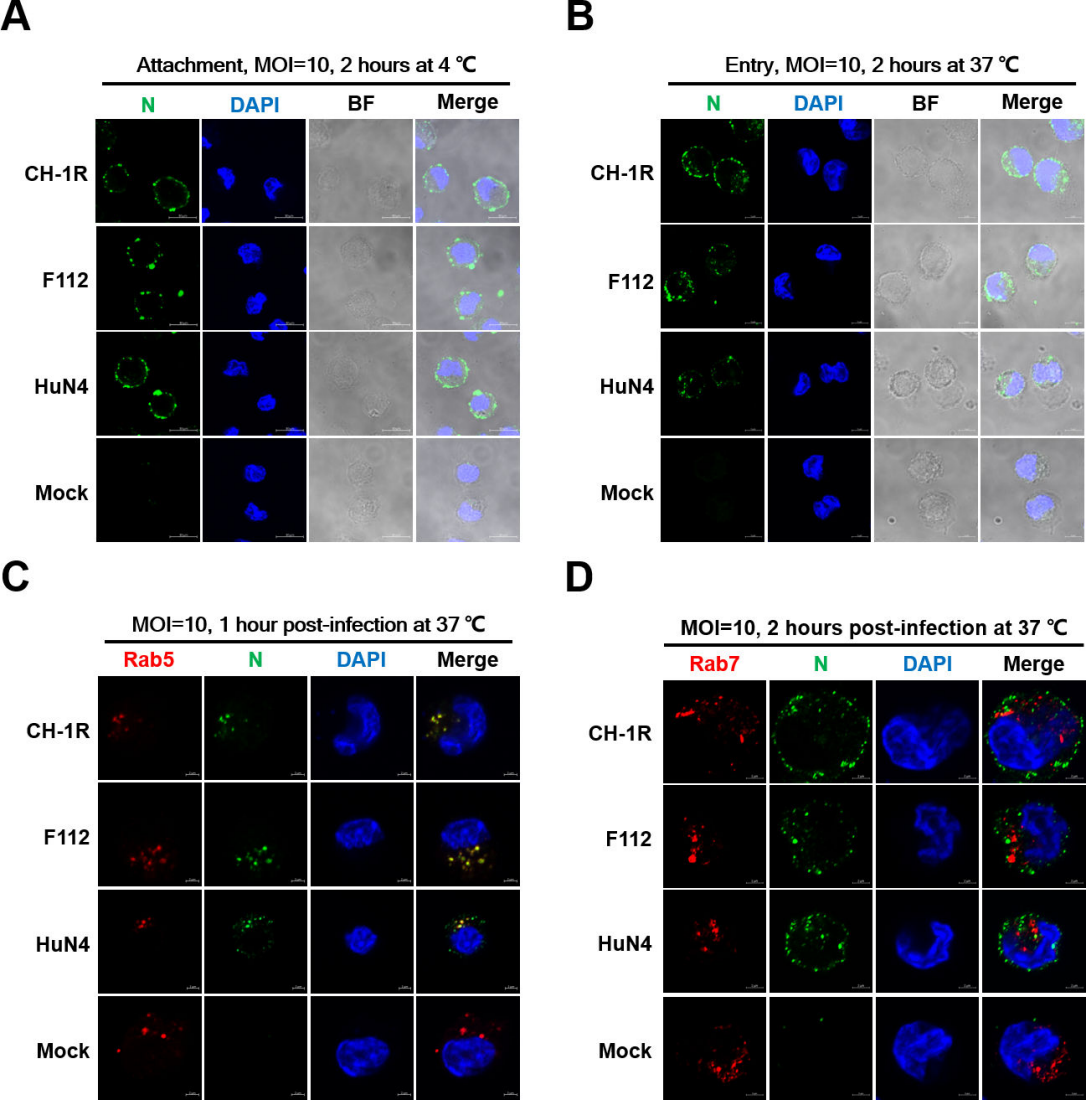
The live attenuated vaccines can attach to and be internalized in primary PAMs. (**A**) Primary PAMs were inoculated with HuN4, F112, or CH-1R (MOI = 10) at 4°C, with mock infection used as the control. After 2 hours, the cells were washed three times with cold PBS to remove the unbound virus. The cells were then fixed and subjected to an IFA with an antibody against PRRSV N protein. Scale bars, 10 µm. (**B**) Primary PAMs were inoculated with HuN4, F112, or CH-1R (MOI = 10) at 4°C for 2 hours. The cells were subsequently washed three times with cold PBS and then supplemented with fresh medium. The cells were maintained at 37°C for 2 hours. The cells were subsequently fixed and subjected to an IFA with an antibody against PRRSV N protein. Scale bars, 5 µm. (**C**) Primary PAMs were inoculated with HuN4, F112, or CH-1R at MOI of 10, with uninfected cells serving as the control. At the indicated times post-inoculation, the cells were fixed, permeabilized, and immunostained for PRRSV N protein and Rab5 or (**D**) Rab7. Scale bars, 2 µm. The experiments were repeated three times independently, and representative data are displayed.

The lifecycle of PRRSV starts with viral attachment, followed by internalization. PRRSV LAVs have undergone serial passages on monkey cells and lost their tropism for primary PAMs. We then attempted to determine whether primary PAMs restrict PRRSV LAV strains at the entry stage. We first evaluated the viral attachment of PRRSV LAVs to primary PAMs. The CH-1R and F112 strains were selected as representative PRRSV LAVs, and HuN4 was used as a control. Primary PAMs were incubated with CH-1R, F112, or HuN4 at MOI of 10 for 2 hours at 4°C. This elevated MOI was employed to enhance the visualization of viral attachment through IFA. The results indicated that both CH-1R and F112 could attach to the cell surface of primary PAMs (Fig. 2A). Next, we evaluated viral internalization by primary PAMs. The primary PAMs were infected with CH-1R, F112, or HuN4 at MOI of 10 at 37°C for 2 hours. The infected primary PAMs were subsequently fixed, and an IFA was performed. We found that, similar to HuN4, both CH-1R and F112 could be internalized into the cytoplasm of primary PAMs (Fig. 2B).

PRRSV requires trafficking through CD163-positive early endosomes for productive infection; however, it does not need to traffic through late endosomes (40). We subsequently investigated whether primary PAMs restrict LAV replication at this stage. Primary PAMs were infected with CH-1R, F112, or HuN4 (MOI = 10) and then fixed at the indicated time points post-infection. Early endosomes (Rab5), late endosomes (Rab7), and lysosomes (Lamp1) were labeled with specific antibodies. The results indicated that CH-1R, F112, and HuN4 were highly colocalized with early endosomes rather than with late endosomes and lysosomes (Fig. 2C and D; Fig. S1). These results suggested that PRRSV LAVs were able to attach to and enter primary PAMs and even enter early endosomes as the virulent HuN4 strain.

### Primary PAMs restrict CH-1R and F112 replication before the viral protein and RNA synthesis stages

**Fig 3:**
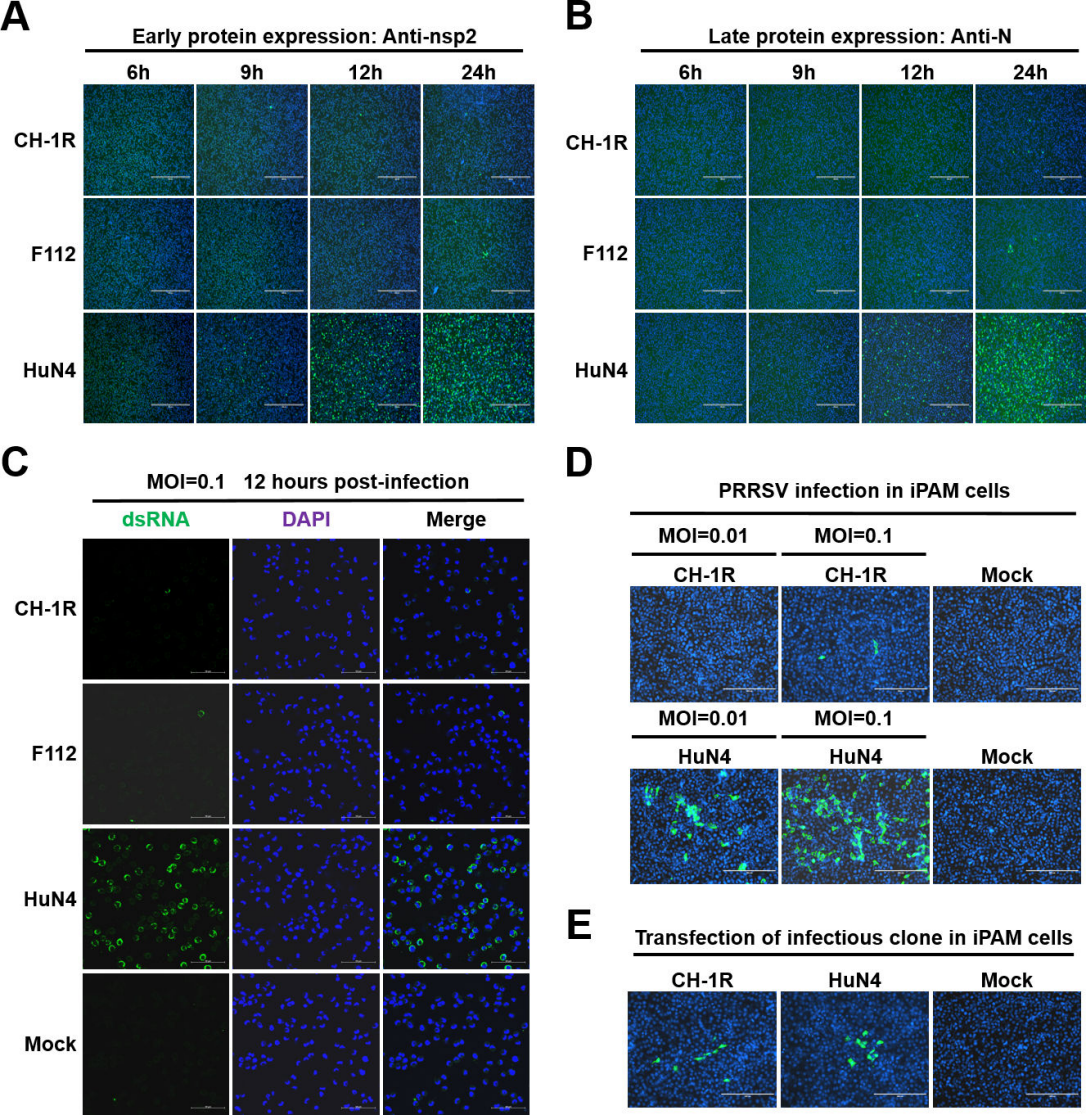
Synthesis of viral protein and RNA of the live attenuated vaccines is defective in primary PAMs. (**A**) Primary PAMs were infected with HuN4, F112, or CH-1R at MOI of 0.1. At 6, 9, 12, and 24 hours post-infection, the cells were fixed and subjected to an IFA with antibodies against PRRSV nsp2 or (**B**) the N protein. Scale bars, 400 µm. (**C**) Primary PAMs were infected with HuN4, F112, or CH-1R at MOI of 0.1. At 12 hours post-infection, the cells were fixed and subjected to an IFA with a dsRNA antibody. Scale bars, 50 µm. (**D**) iPAM cells were infected with HuN4 and CH-1R at the indicated MOI. At 36 hours post-infection, the cells were subjected to an IFA with an antibody against PRRSV N protein. Scale bars, 200 µm. (**E**) iPAM cells were transfected with the indicated PRRSV infectious clone plasmids. At 24 hours post-transfection, the cells were fixed and subjected to an IFA with an antibody against PRRSV N protein. Scale bars, 200 µm.

After virus entry, the viral genome is released, followed by the synthesis of viral proteins and RNA. We therefore tested whether PRRSV LAV protein synthesis is blocked in primary PAMs. We first assessed the expression of an early viral protein, nsp2. Primary PAMs were infected with CH-1R, F112, or HuN4 at MOI of 0.1. At the indicated time points post-infection, the cells were fixed and stained with an anti-nsp2 antibody. For HuN4, nsp2 expression was detected in many cells beginning at 9 hours post-infection, and its expression increased gradually as the infection progressed (Fig. 3A). By contrast, for CH-1R and F112, only sporadic cells expressed PRRSV nsp2 (Fig. 3A). We also detected a late viral protein, the N protein, and obtained a similar result to that for nsp2 (Fig. 3B).

Next, we assessed viral RNA synthesis in primary PAMs. Primary PAMs were infected with CH-1R, F112, or HuN4 at an MOI of 0.1. At 12 hours post-infection, intracellular viral dsRNA was detected via an anti-dsRNA antibody. We found that in HuN4-infected cells, dsRNA could be detected in most primary PAMs. However, in the CH-1R and F112 infection groups, only sporadic cells presented positive dsRNA signals (Fig. 3C). Therefore, we speculated that primary PAMs restricted the replication of PRRSV LAVs before or at the stage of viral protein and RNA synthesis. To confirm this hypothesis, we transfected infectious clones of CH-1R and HuN4 into PAMs, as viral genomic RNA can be transcribed under the CMV promoter (17). However, the transfection efficiency of primary PAMs is extremely low. In our previous work, we immortalized primary PAMs with the SV40 large T antigen and developed an iPAM cell line with higher transfection efficiency that supports PRRSV infection (41). We found that this iPAM cell line mimicked primary PAMs well for CH-1R and HuN4 infections at different infection doses (Fig. 3D). We subsequently transfected cDNA infectious clones of HuN4 and CH-1R into iPAM cells (42). At 24 hours post-transfection, we detected the N protein in cells transfected with both CH-1R and HuN4 (Fig. 3E). These results demonstrated that PAMs restricted the replication of PRRSV LAVs before the stages of viral protein and RNA synthesis.

### PAMs restrict LAV infection at the uncoating step

The restriction of PRRSV LAV infection in primary PAMs occurs after viral entry but before the synthesis of viral proteins and RNA. Upon PRRSV infection, virions enter the early endosome, where fusion between the viral envelope and early endosome takes place, leading to uncoating and release of the viral genome. On the basis of these findings, we proposed that primary PAMs may restrict PRRSV LAVs at the uncoating stage. To test this hypothesis, we employed DiD, a lipophilic dye with self-quenching properties at high concentrations, to investigate virus‒cell membrane fusion. As virus‒cell membrane fusion occurs, DiD diffuses across the host cell membrane, increasing fusion-associated fluorescence (43).

**Fig 4:**
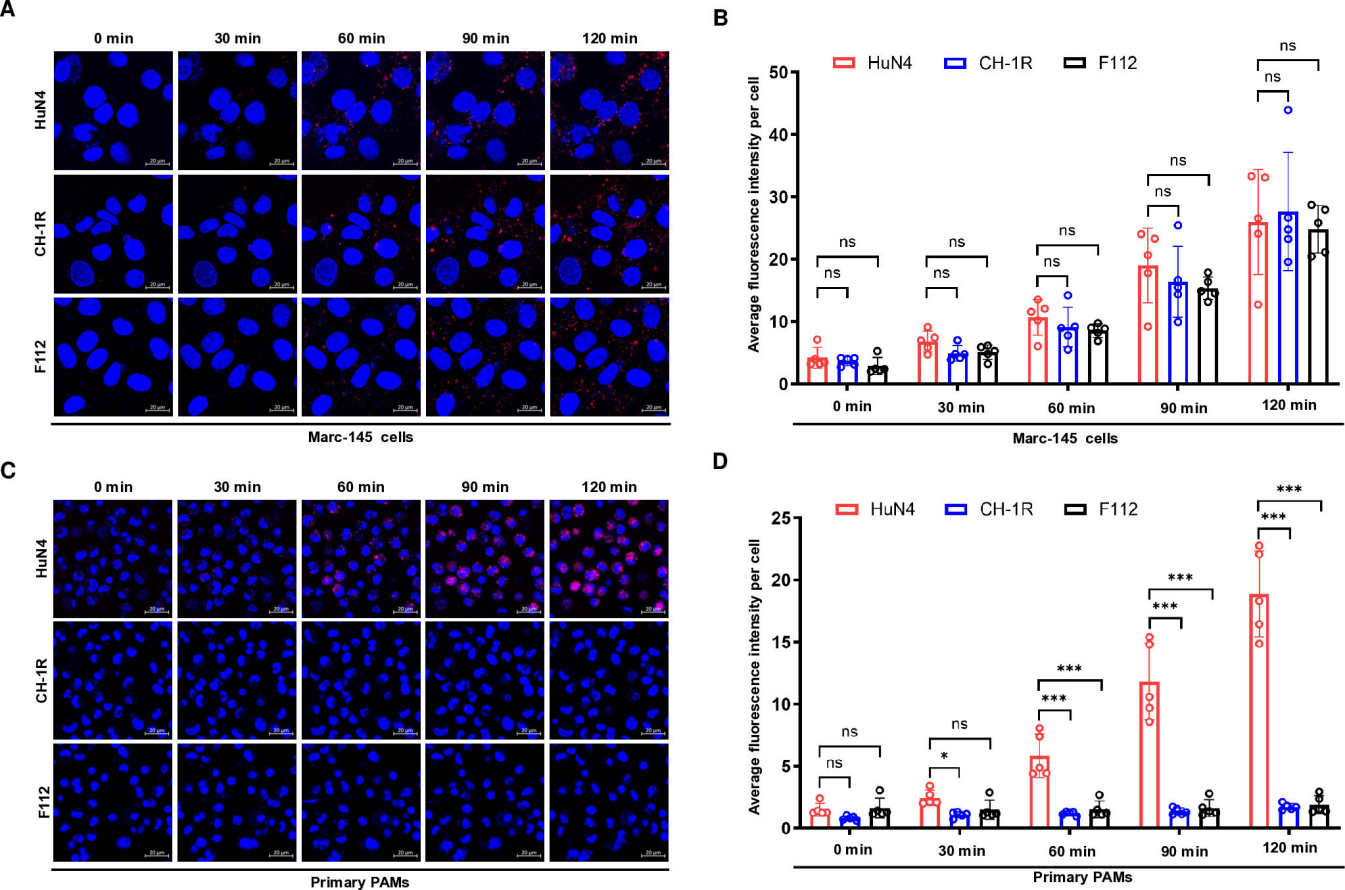
The uncoating step of the live attenuated vaccines is blocked in primary PAMs. (**A**) Marc-145 cells or (**C**) primary PAMs were prestained with DAPI and infected with CH-1R-DiD or HuN4-DiD (MOI = 1) at 4°C for 30 minutes. After incubation, the cells were washed with PBS and then supplemented with new medium. The cells were immediately observed via a live confocal microscope. At the indicated time points post-infection, DiD fluorescence signals were captured. Scale bars, 20 µm. The experiments were independently repeated three times, and representative data are shown. (**B**) The average DiD fluorescence intensity per cell was calculated for Marc-145 cells and (**D**) primary PAMs at specified time points using ImageJ software. Five random fields of view were analyzed at each time point. An asterisk (*) denotes a significant difference between the groups (ns: no significant difference; *P < 0.05; ***P < 0.001).

We initially purified CH-1R and HuN4 virions through density gradient centrifugation with cesium chloride. The purified virions were subsequently labeled with DiD. Marc-145 cells were subsequently infected with the indicated DiD-labeled viruses at 4°C for 30 minutes. The cells were promptly transferred to a confocal microscope, and real-time fluorescence microscopy was used to monitor the intracellular DiD fluorescence signals every 5 minutes. Notably, distinct DiD fluorescence signals were observed in Marc-145 cells infected with HuN4-DiD, F112-DiD, or CH-1R-DiD at 60 minutes post-infection. The fluorescence signal gradually increased with increasing duration of infection (Fig. 4A and B), indicating that HuN4, F112, and CH-1R successfully completed the uncoating process in Marc-145 cells. We subsequently assessed the uncoating of the HuN4-DiD, F112-DiD, and CH-1R-DiD viruses in primary PAMs. Interestingly, while DiD fluorescence signals were observed in primary PAMs infected with HuN4-DiD, the intensity of fluorescence of DiD was increasing with the duration of infection (Fig. 4C and D). However, no such signals were detected in those infected with F112-DiD or CH-1R-DiD (Fig. 4C and D). These findings highlighted a deficiency in viral uncoating for PRRSV LAVs when infecting primary PAMs.

### The nsp2 gene contributes to viral uncoating in primary PAMs

**Fig 5:**
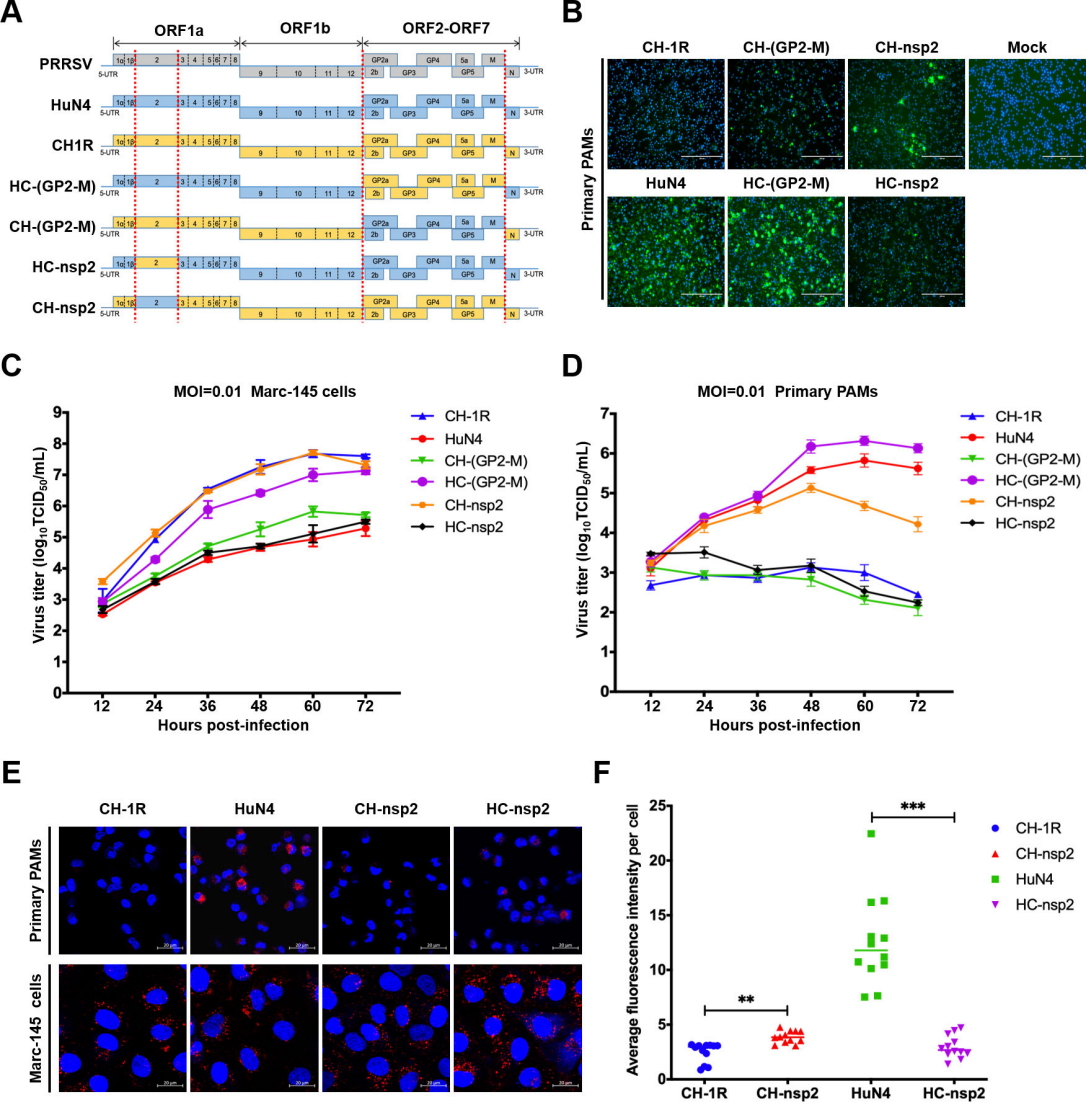
Nsp2 contributes to the uncoating of live attenuated PRRSV in PAMs. (**A**) Schematic diagram of chimeric PRRSVs. (**B**) Primary PAMs were infected with the indicated chimeric PRRSV at MOI of 0.01. At 48 hours post-infection, the cells were fixed and subjected to an IFA with an antibody against PRRSV N. Scale bars, 200 µm. (**C**) Marc-145 cells and (**D**) primary PAMs were infected with chimeric PRRSV mutants at MOI of 0.01. At the indicated time points post-infection, the viruses were harvested and quantified via the TCID50 method. (**E**) Primary PAMs and Marc-145 cells were infected with CH1R-DiD, HuN4-DiD, CH-nsp2-DiD, or HC-nsp2-DiD (MOI = 1) at 4°C for 30 minutes. After incubation, the cells were washed twice with PBS and then supplemented with fresh medium. The cells were subsequently maintained at 37°C. After 2 hours, the cells were fixed and stained with DAPI. Scale bars, 20 µm. (**F**) Primary PAMs were infected with PRRSV-DiD at an MOI of 1. At 2 hours post-infection, the cells were fixed and stained with DAPI. Twelve random fields of view were captured, and the average DiD fluorescence intensity per cell was calculated via ImageJ software. An asterisk (*) indicates a significant difference between the groups (ns: no significant difference; **P < 0.01; ***P < 0.001).

We subsequently identified the genes involved in viral uncoating in primary PAMs. In our laboratory, we possess two cDNA infectious clones, namely, CH-1R and HuN4. Therefore, leveraging these two infectious clones as a framework, we strategically replaced the corresponding regions, as depicted in Fig. 5A. We first replaced the viral structural genes, from GP2 to M, which have been reported to be associated with viral tropism and spreading patterns (17, 44). The chimeric viruses CH-(GP2-M) and HC-(GP2-M) were successfully rescued in Marc-145 cells (Fig. S2). Surprisingly, the structural genes did not contribute as expected to PRRSV LAV tropism in primary PAMs (Fig. 5B). Viral proteins involved in PRRSV uncoating should be present in the virions. It has been previously reported that nsp2 is associated with PRRSV virions (45). Therefore, we aimed to investigate the potential contribution of nsp2 to viral uncoating by generating chimeric viruses with replaced nsp2. In primary PAMs, the replacement of CH-1R nsp2 with HuN4-derived nsp2 partially restored the replication ability of CH-nsp2, whereas the substitution of HuN4 nsp2 with CH-1R-derived nsp2 resulted in the near complete failure of replication of HC-nsp2 (Fig. 5B). We also assessed the replication kinetics of chimeric viruses and parental viruses in Marc-145 cells and primary PAMs. The replication capacity was severely impaired for HC-nsp2 and CH-(GP2-M) in primary PAMs; however, efficient replication was observed for CH-nsp2 and HC-(GP2-M) in primary PAMs, as evidenced by the viral titers (Fig. 5C and D).

We subsequently conducted additional experiments to investigate the correlation between nsp2 and viral uncoating in primary PAMs. Chimeric viruses of either CH-nsp2 or HC-nsp2 were purified and labeled with DiD dye. Following infection of primary PAMs with the labeled viruses, Marc-145 cells were used as a positive control. At 2 hours post-infection, fluorescence signals were observed in the CH-nsp2-infected PAMs, which were significantly more intense than those in the CH-1R-infected PAMs. Conversely, the fusion fluorescence signal intensity was notably lower in the HC-nsp2 group than in the HuN4 group (Fig. 5E and F). Consequently, our findings suggested that nsp2 contributed to the viral uncoating process within primary PAMs.

### The pathogenicity of the chimeric HC-nsp2 virus in piglets is attenuated

**Fig 6:**
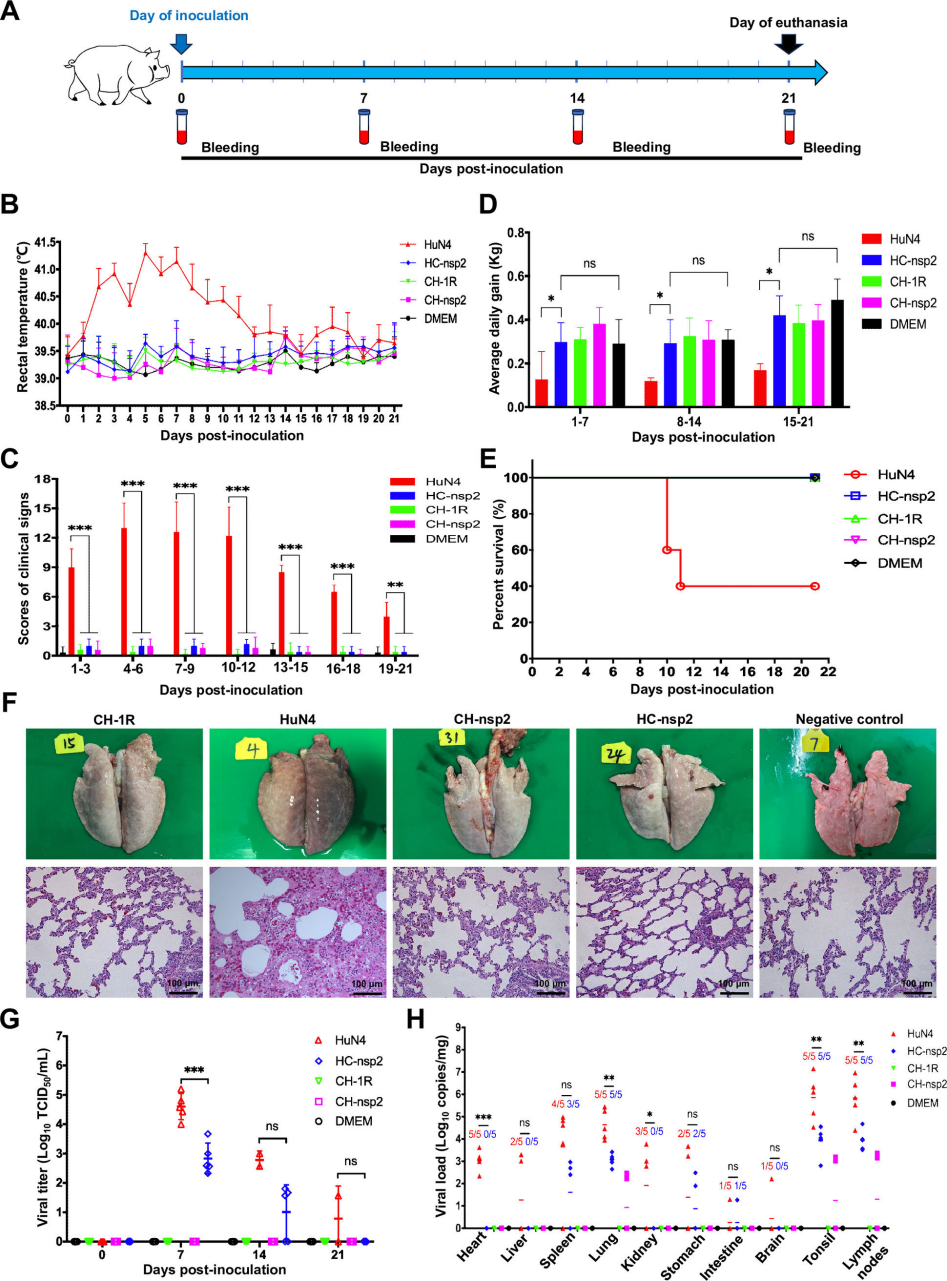
Pathogenicity of chimeric viruses. (**A**) Scheme of the animal experimental protocol. (**B**) Rectal temperatures of the piglets in each group were determined daily. A rectal temperature ≥40.5°C was defined as fever. (**C**) The clinical scores of piglets in each group after virus inoculation were evaluated. The clinical scoring system involved evaluating the gross clinical score, respiratory clinical score, and nervous signs score. (**D**) The average daily body weight gain of the piglets in each group was evaluated. (**E**) Analysis of animal survival curves. (**F**) Representative gross lung lesions and microscopy images of H&E-stained lung lesions in each group are presented. Scale bars, 100 µm. (**G**) The viral load in the serum of individual piglets at different time points post-inoculation was quantified in Marc-145 cells via the TCID50 method. (**H**) The viral loads in 10 tissues were determined by RT-qPCR. The numbers indicate the count of piglets with detectable viral loads and the total number of piglets in each group. All the data are presented as the means and standard deviations (error bars). An asterisk (*) indicates a significant difference between the groups (ns: no significant difference; *P < 0.05; **P < 0.01; ***P < 0.001).

Given the association of nsp2 with viral replication in primary PAMs, we subsequently investigated its potential contribution to the pathogenicity of PRRSV *in vivo*. To assess this, we evaluated the pathogenicity of the chimeric viruses HC-nsp2 and CH-nsp2 in piglets, utilizing CH-1R and HuN4 as controls for the vaccine strain and virulent strain, respectively. A total of twenty-three 28-day-old piglets were randomly divided into five groups (each with five pigs, whereas the negative control group consisted of three piglets). All piglets in each group were intramuscularly injected with a dose of 2 × 10^5.0^ TCID50 per pig for the CH-1R, HuN4, CH-nsp2, or HC-nsp2 strains. The negative control group was inoculated with DMEM, and samples were collected as illustrated in Fig. 6A. Daily monitoring of rectal temperatures and clinical symptoms was conducted on all piglets. In the HuN4-inoculated group, persistent fever was recorded, with the body temperature exceeding 40.5°C for 6 days, and the highest recorded temperature was 41.6°C (Fig. 6B). Conversely, the piglets inoculated with HC-nsp2 did not exhibit any noticeable clinical symptoms or fever, resulting in significantly lower clinical scores than those of the HuN4-inoculated group from days 1 to 21 post-infection (Fig. 6B and C). Furthermore, the average daily weight gain of piglets in the HC-nsp2-inoculated group exceeded that of those in the HuN4 group (Fig. 6D). In addition, the clinical scores of HuN4-inoculated piglets were significantly elevated. The HuN4-inoculated piglets presented severe clinical symptoms, including coughing, listlessness, recumbency, and anorexia (Fig. 6C). By contrast, piglets in the CH-1R-inoculated group, CH-nsp2-inoculated group, and negative control group did not display any discernible clinical signs of PRRSV infection, and there were no significant differences in terms of clinical scores or daily body weights among the three groups (Fig. 6C and D). Three piglets died in the HuN4-inoculated group from day 10 to day 11 after inoculation, whereas no mortalities occurred in the other groups (Fig. 6E). The piglets in the HuN4-inoculated group presented characteristic PRRS lesions, including lung consolidation, congestion, and hemorrhaging in the submaxillary lymph nodes (Fig. 6F; Fig. S3). However, no significant histopathological changes were observed in the piglets in the other groups (Fig. 6F). The HuN4-inoculated group also had significantly higher pulmonary lesion score than the HC-nsp2-inoculated group as well as the other groups (Fig. S4). We subsequently assessed the viremia levels among the different experimental groups. Serum samples were collected at specified time points and quantified via a TCID50 assay. The HuN4-inoculated group presented high viremia levels in all the piglets (Fig. 6G). However, the HC-nsp2-inoculated group presented significantly lower viremia levels than did the HuN4-inoculated group. No infectious virus was detected in the CH-nsp2-inoculated group, CH-1R-inoculated group, or negative control group (Fig. 6G). In addition, real-time PCR analysis revealed that viral loads in specific organs, such as the heart, lung, tonsils, and lymph nodes, were significantly lower in the HC-nsp2-inoculated group than in the HuN4-inoculated group (Fig. 6H). These findings suggested a strong correlation between nsp2 and PRRSV pathogenicity in piglets.

### Immunization with HC-nsp2 confers protection against HuN4 challenge

**Fig 7:**
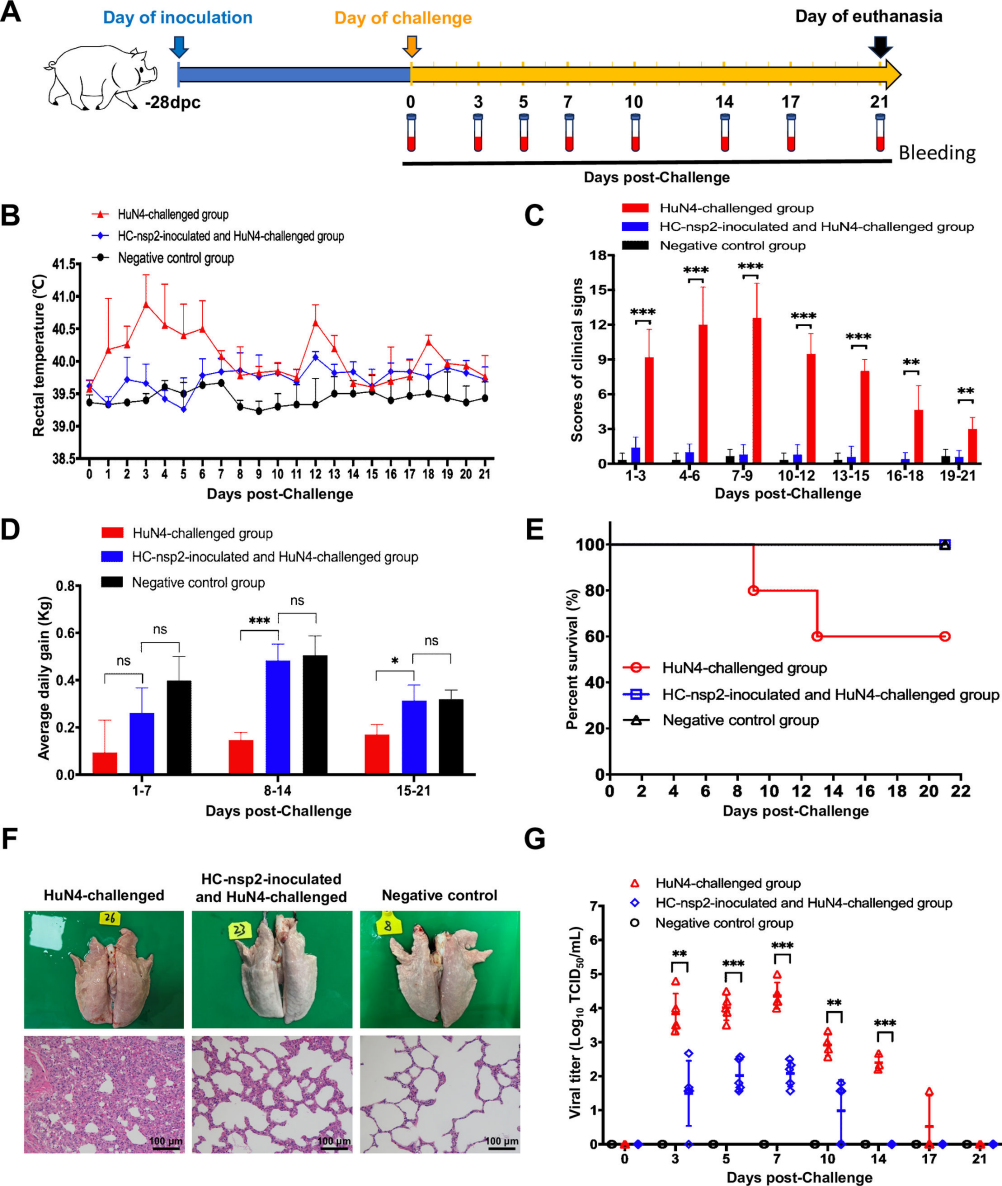
HC-nsp2 provides clinical protection against HuN4 challenge. (**A**) Scheme of the animal experimental protocol. (**B**) Rectal temperatures of the piglets in each group were determined daily. A rectal temperature ≥40.5°C was defined as fever. (**C**) The clinical scores of the piglets in each group after the HuN4 challenge were evaluated. The clinical scoring system involved evaluating the gross clinical score, the respiratory clinical score, and the nervous system signs score. (**D**) Average daily body weight gains of piglets in each group after the HuN4 challenge were evaluated. (**E**) Analysis of animal survival curves. (**F**) Representative gross lung lesions and microscopy images of H&E-stained lung lesions in each group are presented. Scale bars, 100 µm. (**G**) The viral load in the serum of individual piglets at different time points post-challenge was determined in Marc-145 cells via the TCID50 method. All the data are presented as the means and standard deviations (error bars). An asterisk (*) indicates a significant difference between the groups (ns: no significant difference; *P < 0.05; **P < 0.01; ***P < 0.001).

Next, we assessed the potential of HC-nsp2 as a vaccine candidate that could confer protection against HuN4 challenge. Each group of piglets received individual intramuscular injections of either HC-nsp2 or DMEM, with a viral dosage of 2×10^5.0^ TCID50 per pig. At 28 days post-inoculation, the piglets were challenged with 2×10^5.0^ TCID50 of HuN4 per pig (Fig. 7A). We found that the HC-nsp2-inoculated group did not exhibit any noticeable fever or clinical symptoms after challenge with HuN4 (Fig. 7B and C). Furthermore, the HC-nsp2-inoculated group presented greater weight gain (Fig. 7D). Two piglets died in the HuN4-challenged group, whereas no deaths occurred in the inoculated and challenged groups (Fig. 7E). Furthermore, no significant histopathological changes were observed in the piglets in the HC-nsp2-inoculated group after challenge with HuN4 (Fig. 7F; Fig. S5). In addition, the piglets in the HC-nsp2-inoculated group had lower pulmonary lesion scores after challenge with HuN4 (Fig. S6). Viremia levels were subsequently assessed, and the inoculation and challenge groups presented significantly lower viremia levels than the HuN4-challenged group did (Fig. 7G). Overall, these results indicated that HC-nsp2 provided protection against the highly virulent HuN4 challenge.

## DISCUSSION

Viral infections pose a significant threat to the health of both humans and animals. LAVs remain the most effective and widely used tool for combating viral infections. However, until now, the underlying mechanisms responsible for attenuation in most LAVs, including the PRRSV-2 LAVs, have remained elusive. PRRSV infection is predominantly restricted to cells of the monocyte‒macrophage lineage, including PAMs (46, 47), as well as macrophages derived from the spleen, tonsils, lymph nodes, liver, thymus, and peritoneal macrophages from the bone marrow and blood (48, 49). Among these cell types, PAMs serve as the primary target for PRRSV infection *in vivo*. A recent study demonstrated a positive correlation between the replication efficiency of PRRSV in PAMs and its virulence *in vivo* (39). Most commercially available PRRSV LAVs have been developed through the successive passage of virulent strains on monkey kidney cells such as Marc-145 cells. In this study, we observed robust replication of five commercial PRRSV LAVs in Marc-145 cells; however, their replication in PAMs was significantly restricted (Fig. 1). Viral attachment and internalization are the first processes by which PRRSV infects target cells. Our results suggested that LAVs have the ability to attach and internalize in primary PAMs (Fig. 2). In addition, we observed that PRRSVs could enter early endosomes but did not continue through the endocytic pathway to late endosomes or lysosomes, as shown in a previous study (Fig. 2) (40). We further discovered that LAVs presented a replication defect prior to viral RNA and protein synthesis in primary PAMs (Fig. 3). Ultimately, the replication defects of LAVs in primary PAMs were specifically in the viral uncoating stage (Fig. 4). Previous studies have highlighted the importance of PRRSV minor glycoproteins (GP2a, GP3, and GP4) as crucial determinants of viral tropism in PAMs (50, 51). Furthermore, our recent findings also demonstrated that the GP2a-GP4 region contributes to both the yield and spreading patterns of PRRSV in Marc-145 cells (17). The uncoating process of PRRSV occurs in the early stages of viral infection and is typically mediated by virion-associated proteins. However, how nsp2 regulates PRRSV uncoating is still unclear. CD163, an important receptor for PRRSV, is involved mainly in the process of viral particle uncoating. Multiple envelope proteins of PRRSV can interact with CD163 (52, 53). Further research is needed to determine whether nsp2 influences viral uncoating by regulating viral envelope proteins. Our recent study showed that the full-length nsp2 replicase plays a role in viral assembly in highly pathogenic PRRSV-2 (38).

Notably, nsp2, the largest cleavage product of the PRRSV replicase, is also encapsulated within virions (45). Therefore, in this study, we investigated the contribution of nsp2 to the uncoating process of CH-1R in primary PAMs, and our findings revealed that nsp2 significantly influenced the cellular tropism of CH-1R on PAMs (Fig. 5). In this study, we swapped the full-length nsp2 between CH-1R and HuN4 and demonstrated that nsp2 contributed to viral tropism in PAMs *in vitro* and virulence *in vivo*. However, it remains unclear whether other nsp2 isoforms also contribute to these phenotypes. We aim to determine which domain is responsible for this phenotype in future investigations. Indeed, the nsp2 nucleic acid sequences of HuN4 and CH-1R exhibited significant differences, with only 92.75% nucleic acid identity, leading to 154 amino acid mutations (Fig. S7). Unraveling which specific nsp2 amino acids contribute to viral tropism in PAMs *in vitro* and virulence *in vivo* presents a considerable challenge. In addition, the nsp2 sequence differences between HuN4 and CH-1R may result in different nsp2 isoforms, potentially affecting virus uncoating. In a previous study by Song et al., the highly variable region of nsp2 determined viral cellular tropism for another HP-PRRSV JXwn06 strain (25). However, our study differs from theirs. Song et al. used deletion to identify the critical region for PRRSV tropism in PAMs and demonstrated that the identified region influenced both viral subgenomic mRNA synthesis and viral tropism. The deletion mutants they identified also presented significantly decreased titers in Marc-145 cells; however, in our study, the CH-1R titer was higher than that of HuN4 in Marc-145 cells, and the replacement of nsp2 did not influence the virus titer in Marc-145 cells (Fig. 5).

Currently, researchers are actively investigating key virulence genes that influence the pathogenicity of PRRSV. Through gene substitution experiments involving the JXwn06 and HB-1/3.9 strains, Li et al. reported a close association between nsp9 and nsp10 and PRRSV pathogenicity (54). In a recent study, Kong et al. reported that nsp2 was a crucial virulence factor of PRRSV, emphasizing the importance of genetic variation in nsp2 in regulating PRRSV virulence and persistence (24). However, our study differed from their work in several ways. First, in their study, they used a low-virulence NADC30-like strain, CHsx1401, as the parental strain and replaced its nsp2 and structural protein-coding regions with those of the HP-PRRSV strain JXwn06 to investigate key genes influencing PRRSV pathogenicity. However, in contrast to CH-1R and other commercial vaccine strains, low-virulence CHsx1401 could replicate well in PAMs. This finding indicated that nsp2 of low-virulence CHsx1401 did not influence virus uncoating in PAMs. Therefore, nsp2 may have different mechanisms to determine PRRSV virulence. Second, our results were more convincing than those of the study by Kong et al., as they failed to successfully rescue a chimeric virus in which only the nsp2 region was swapped. They reported that swapping both nsp2 and structural proteins from the JXwn06 strain conferred enhanced pathogenicity to the CHsx1401 strain. By contrast, in our study, we successfully rescued a chimeric virus in which only the nsp2 was replaced. This finding provides direct evidence that nsp2 contributes to the virulence of PRRSV. In our study, we observed that nsp2 from the attenuated CH-1R strain was sufficient to attenuate the virulence of the HP-PRRSV HuN4 strain. HC-nsp2 infection did not induce high fever or other typical clinical signs associated with HP-PRRS throughout the infection period (Fig. 6B and C). The replacement of the nsp2 gene provides a plausible explanation for the observed differences in replication within PAMs. However, the *in vivo* data do not fully align with the effects attributed to the nsp2 gene swap. For instance, as shown in Fig. 6G, CH-nsp2 exhibited a phenotype similar to CH-1R and was undetectable in the blood. In other organs, the nsp2 gene appeared to only partially modify the viral phenotype. This phenomenon can be attributed to the fact that the virulence of CH-1R, a strain derived from a highly adapted Marc-145 cell line, is likely influenced by multiple genetic factors (17). Indeed, viral virulence is multifactorial, encompassing various processes such as virus attachment, internalization, uncoating, viral RNA synthesis, and release during the replication cycle (3). Our results indicated that the replacement of the nsp2 in HuN4 with that from CH-1R significantly impacted the uncoating process of the chimeric virus in PAMs. This finding suggested that the nsp2 from CH-1R played a crucial role in determining whether PRRSV can undergo normal uncoating in PAMs. However, in Fig. 5E and F, although the average intensity of CH-nsp2 is higher than that of CH-1R, it is significantly less than that of HuN4, suggesting that nsp2 does not completely restore the signal to HuN4’s level and that other viral genes may also influence the viral uncoating process. However, it is important to note that CH-1R has undergone 160 passages in Marc-145 cells, where numerous genes may have experienced changes affecting viral replication and viral uncoating. Therefore, this study demonstrates that other viral genes may also be involved in uncoating efficiency. In future, the replacement of additional related genes will help us identify additional attenuation mechanisms of CH-1R. Furthermore, the use of PRRSV strains of low to moderate virulence as recipients of HuN4 nsp2 could be a possible alternative to observe obvious phenotypic changes in virulence. This finding suggested that nsp2 did not solely determine the virulence of the CH-1R virus; further investigations are needed to explore potential associations between viral nsp2 and other replicative enzymes or envelope proteins in determining viral pathogenicity.

PRRSV LAVs have played a crucial role in the prevention and control of PRRS outbreaks since the initial occurrence of PRRS in the late 1980s (55). While these vaccines offer effective immune protection against closely related strains, their efficacy against genetically diverse viruses is limited. Given the propensity of PRRSV for genetic recombination and mutation, the emergence of NADC30-like and NADC34-like strains followed epidemics of classic PRRSV and HP-PRRSV in China (16, 56). The traditional methods for LAV development are time-consuming, labor-intensive, and susceptible to contamination during passaging. Therefore, establishing a rapid virus attenuation strategy capable of generating modified live vaccine candidates from various PRRSV lineages to effectively combat future severe PRRS outbreaks is imperative.

Overall, the present study offers novel insights into the attenuation mechanism of PRRSV LAVs and demonstrates the feasibility of a rapid attenuation strategy for PRRSV. These findings also provide a valuable theoretical foundation for future advancements in PRRSV vaccine development.

## MATERIALS AND METHODS

### Cells, viruses, and antibodies

Marc-145 cells were cultured in Dulbecco’s modified Eagle’s medium (DMEM) (Gibco, USA) supplemented with 10% fetal bovine serum (FBS, Excel, FND500) at 37°C with 5% CO2_2_. Primary porcine alveolar macrophages (PAMs) were obtained from 30-day-old SPF pigs and cultured in RPMI 1640 (Gibco, USA) supplemented with 10% fetal bovine serum at 37°C with 5% CO2. iPAM cells were cultured as described previously (41). The highly virulent Chinese PRRSV strain HuN4 was rescued from a PRRSV HuN4 infectious clone (PRRSV HuN4-F5) (42). The PRRSV vaccine strain CH-1R was rescued from the PRRSV CH-1R infectious clone (17). The PRRSV vaccine strain F112 (HuN4-F112) was stored in our laboratory. The PRRSV vaccine strains GDr180 and JXA1-R were purchased from Guangdong Winsun Bio Pharmaceutical Co., Ltd. (WINSUN BIO, China). The PRRSV vaccine strain Ingelvac PRRS MLV was purchased from Boehringer Ingelheim (Germany). Rabbit anti-Rab5, anti-Rab7, and anti-Lamp1 antibodies were purchased from Proteintech (Proteintech, China). The anti-dsRNA antibody was purchased from Scicons (J2; English and Scientific Consulting Bt., Hungary). Alexa Fluor 488-conjugated goat anti-mouse IgG (H + L) and Alexa Fluor 647-conjugated goat anti-rabbit IgG (H + L) were purchased from Invitrogen (Invitrogen, USA). Mouse monoclonal antibodies specific for the PRRSV nsp2 and N proteins were prepared in our laboratory.

### Construction of chimeric PRRSVs

The highly virulent PRRSV strain HuN4 infectious clone (PRRSV HuN4-F5) and PRRSV CH-1R infectious clone were stored in our laboratory (17, 42). Full-length HuN4 and CH-1R infectious clones were utilized as backbones to exchange the ORF2-ORF6 region or the nsp2 gene region between HuN4 and CH-1R, as described in our previous studies (42, 44). Then, the chimeric viruses were rescued as follows: Marc-145 cells were seeded on six-well plates and transfected with infectious cDNA clone plasmids via X-tremeGENE HP DNA Transfection Reagent according to the manufacturer’s instructions when the cells reached 70%–80% confluence. The rescued viruses were confirmed via an indirect IFA and further verified by sequencing.

### Viral growth kinetics assay

Marc-145 cells or primary PAMs were infected with the indicated viruses (MOI = 0.01) at 4°C, and after 2 hours, the cells were washed three times with PBS to remove the unbound viruses. The cells were subsequently supplemented with fresh medium containing 2% FBS. The viruses were harvested at the indicated times post-infection and stored at −80°C. The virus samples were subsequently titrated on Marc-145 cells via the TCID50 method.

### Immunofluorescence assay

Marc-145 cells or primary PAMs were infected with the indicated viruses. At the indicated times, the cells were fixed with 4% paraformaldehyde (PFA) for 15 minutes at room temperature and washed twice with PBS as described in our previous studies (57–59). Then, the cells were permeabilized with 0.5% Triton X-100 for 10 minutes at 4°C and washed twice with PBS. Next, the cells were blocked with PBS containing 2% bovine serum albumin (BSA) (Coolaber, China) for 30 minutes at 37°C and washed three times with PBS. The cells were subsequently incubated with specific primary antibodies at 37°C for 2 hours and washed three times with PBS. The cells were then incubated with appropriate secondary antibodies at 37°C for 1 hour and washed three times with PBS. The cell nuclei were stained with 4′,6-diamino-2-phenylindole (DAPI) for 15 minutes at 37°C and washed three times with PBS. Finally, the fluorescence signals were detected via an LSM 980 Zeiss confocal microscope.

### Attachment and internalization assay

Primary PAMs were seeded in 24-well plates. Twelve hours later, the cells were infected with CH-1R, F112, or HuN4 (MOI = 10) at 4°C for 2 hours. Then, the cells were washed three times with cold PBS and subsequently fixed, permeabilized, and blocked. The cells were immunostained with a PRRSV N MAb, and an Alexa Fluor 488-conjugated goat anti-mouse IgG (H + L) was used. Nuclear DNA was stained with DAPI. Finally, viral attachment was visualized via a confocal microscope.

To detect virus internalization, primary PAMs were prechilled at 4°C for 15 minutes and then infected with the indicated viruses (MOI = 10) at 4°C for 2 hours. The inoculum was subsequently removed, and then the cells were supplemented with fresh medium and maintained at 37°C for 2 hours. After 2 hours, the cells were subjected to an immunofluorescence assay.

### DiD labeling

The first step involved harvesting and concentrating PRRSV stocks via ultracentrifugation at 40,000 rpm for 3 hours at 4°C. The resulting precipitate was then resuspended in dye buffer and incubated with the dye DiD for 2 hours with gentle vortexing in the dark at room temperature, according to the instructions provided by the manufacturer (Beyotime, China). Free DiD was removed by ultracentrifugation at 30,000 rpm for 16–18 hours through a CsCl gradient of 50% (wt/vol) in an SW41Ti rotor. The visible virus band was collected and further concentrated and purified through ultracentrifugation at 28,800 rpm for 4 hours at 4°C in an SW32Ti rotor. The labeled virions were then filtered through a 0.22 µm syringe filter and stored at −80°C.

### Live-cell imaging of viral uncoating

Marc-145 cells or primary PAMs were grown to approximately 100% confluence in a confocal dish. The cell nuclear DNA was stained with NucBlue Live Ready Probes (Invitrogen, USA) for 20 minutes at 37°C. The cells were then prechilled for 15 minutes at 4°C and incubated with the indicated DiD viruses at MOI of 1 for 30 minutes at 4°C. After incubation, the cells were washed three times with cold PBS to remove the unbound viruses and subsequently supplemented with fresh medium containing 2% FBS, after which the cells were immediately placed under a confocal microscope. The stage of the confocal microscope was maintained at 37°C with 5% CO2 for the cells. The DiD fluorescence signals were captured via an LSM 800 Zeiss confocal microscope. Six random fields of view were selected, and each field was photographed every 5 minutes. The images were processed via ZEN software.

### Animal experiments to determine the pathogenicity of the chimeric virus

Twenty-three 28-day-old piglets were acquired from a commercial pig herd in Harbin that was free of PRRSV, ASFV, PCV2, PRV, and CSFV. The piglets were randomly divided into five groups (Table S2). All piglets in each group were inoculated with the indicated viruses via intramuscular injection, and the dosage was 2×10^50^ TCID50 per pig, as described in Table S2. The entire animal experiment lasted for 21 days. All surviving piglets were euthanized and autopsied on the final day of the experiment. The animals in each group were housed in individual biosafety rooms, with daily records of any clinical signs and rectal temperatures recorded. The clinical symptoms were scored, and the scoring standard was described in a previous study (54). Moreover, the piglets’ body weights were monitored weekly, and blood samples were collected at 0, 7, 14, and 21 days postinoculation.

### Animal challenge experiments

Thirteen 28-day-old piglets were obtained from a commercial pig herd in Harbin that was free of PRRSV, ASFV, PCV2, PRV, and CSFV. The piglets were then randomly allocated into three groups (Table S3). In the HuN4-challenged group, five piglets were inoculated with DMEM and then challenged with HuN4. In the HC-nsp2-inoculated and HuN4-challenged groups, five piglets were inoculated with HC-nsp2 and subsequently challenged with HuN4. The remaining three piglets in the negative control group were designated negative controls. All pigs in each group were inoculated and challenged via intramuscular injection, with the dosages detailed in Table S3. The entire animal experiment lasted for 49 days, and all surviving piglets were euthanized and autopsied at 21 days post-challenge (dpc). The animals in each group were housed in separate biosafety rooms, where daily records were kept for any clinical signs or rectal temperatures. The piglets’ clinical symptoms were scored. Furthermore, the piglets’ body weights were measured weekly, and blood samples were periodically collected from individual piglets at 0, 3, 5, 7, 10, 14, 17, and 21 dpc.

### Assessment of viremia and histological examination

Blood samples were periodically collected from individual piglets at 0, 7, 14, and 21 days post-infection (dpi) and at 0, 3, 5, 7, 10, 14, 17, and 21 days post-challenge (dpc) to test for the presence of viremia. Samples of 10 tissues, including heart, liver, spleen, lung, kidney, lymph node, tonsil, small intestine, brain, and stomach samples, were collected for viral detection via TaqMan-based real-time fluorescence quantitative reverse transcription polymerase chain reaction (RT-qPCR), as described in a previous study (60). At necropsy, the lungs and lymph nodes were collected and subjected to histopathological examination following hematoxylin and eosin (H&E) staining, as detailed in a previous study (61). The histopathological lung lesions were scored, and the scoring standard was described in a previous study (54).

### Statistical analysis

Statistical analysis was conducted via GraphPad Prism 8.0 software. Statistical significance was analyzed via t tests. A *P* value < 0.05 was considered statistically significant.

## Data Availability

All data pertinent to this work are contained within this article and the supplemental material.

## SUPPLEMENTAL MATERIAL

Supplemental material jvi.00636-25-s0001.docx Figures S1 to S7; Tables S1 to S3.

ASM does not own the copyrights to Supplemental Material that may be linked to, or accessed through, an article. The authors have granted ASM a non-exclusive, world-wide license to publish the Supplemental Material files. Please contact the corresponding author directly for reuse.
