# Supplementary material for: Nsp2 replicase-mediated viral uncoating in porcine alveolar macrophages contributes to the attenuation of PRRSV-2 live attenuated vaccine
Source: J Virol. 2025 Aug 4;99(9):e00636-25. doi: 10.1128/jvi.00636-25 (PMC12456151; doi:10.1128/jvi.00636-25)
Supplement: Supplemental material — Figures S1 to S7; Tables S1 to S3. [file jvi.00636-25-s0001.docx]

**Supporting Information**


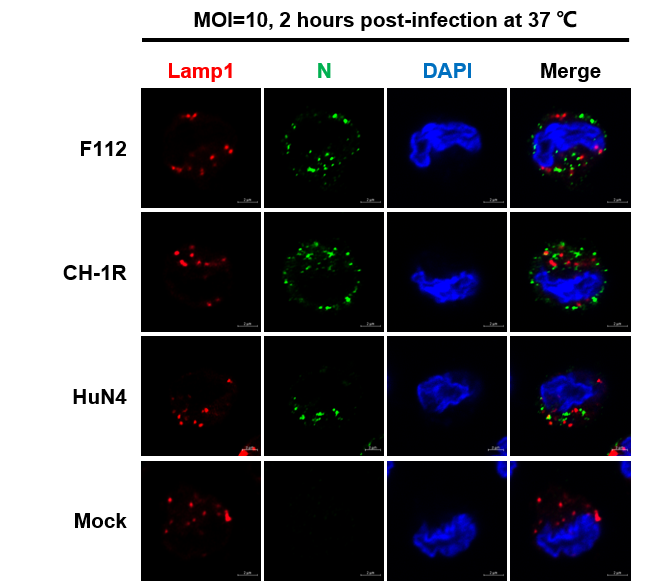


**Figure S1. PRRSV LAVs failed to enter lysosomes in primary PAMs.** Primary PAMs were infected with HuN4, F112, and CH-1R at an MOI of 10, with uninfected cells as the control. After 2 hours post-infection, the cells were fixed and immunostained for PRRSV N and Lamp1. Nuclear DNA was stained with DAPI. The fluorescent signals were observed by confocal microscope. Scale bars, 2 μm. The experiments were independently repeated three times, and representative data were shown.

**
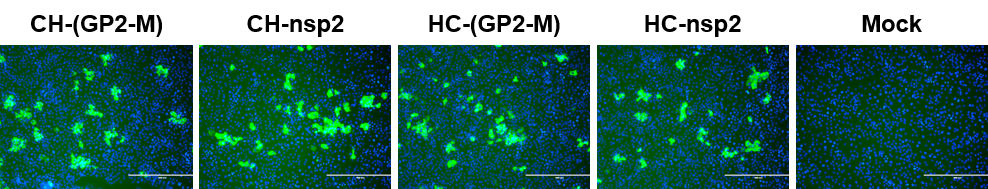
Figure S2. Recovery of chimeric viruses.** Marc-145 cells were transfected with indicated infectious cDNA clone plasmids. The whole cell culture was harvested at 5 days post-transfection and inoculated on the new Marc-145 cells. At 4-5 days post-inoculation, the cells were fixed, permeabilized, and immunostained for PRRSV N MAbs. Scale bars, 400 μm.


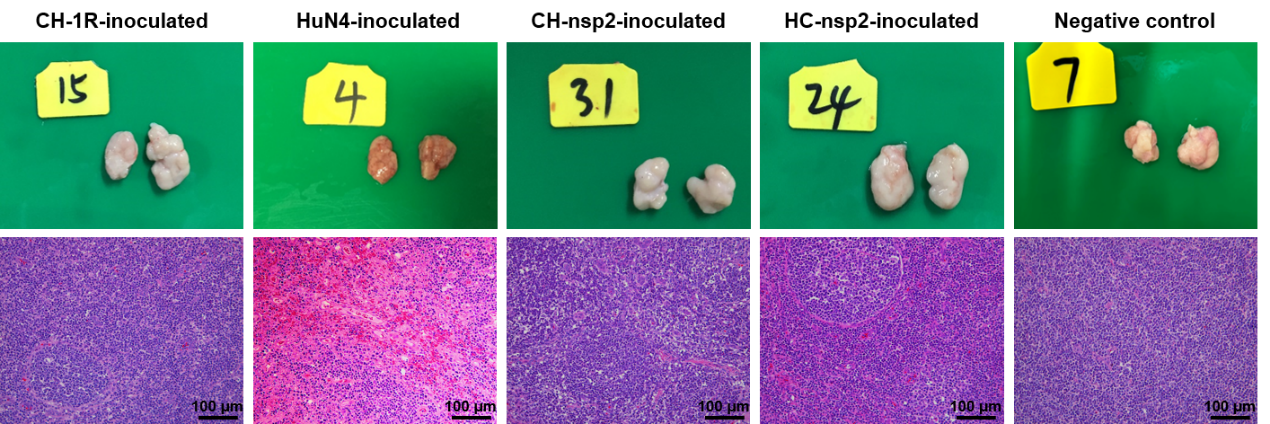


**Figure S3.** **The lymph nodes damage caused by the chimeric viruses.** Representative gross submaxillary lymph nodes, and microscopic submaxillary lymph nodes lesions by H&E staining in each group were presented. Scale bars, 100 μm.


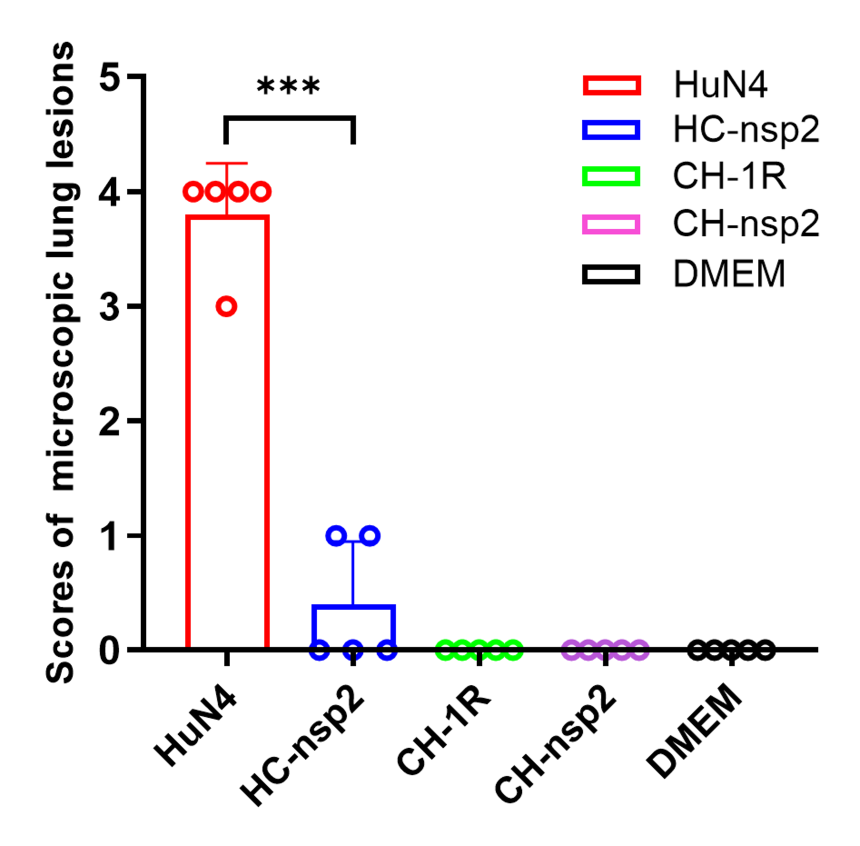


**Figure S4. The pulmonary lesion score of piglets** **in each group after chimeric virus infection.** The histopathological lung lesions of all piglets in each group were scored. An asterisk (*) indicates a significant difference between the groups (***: p < 0.001).


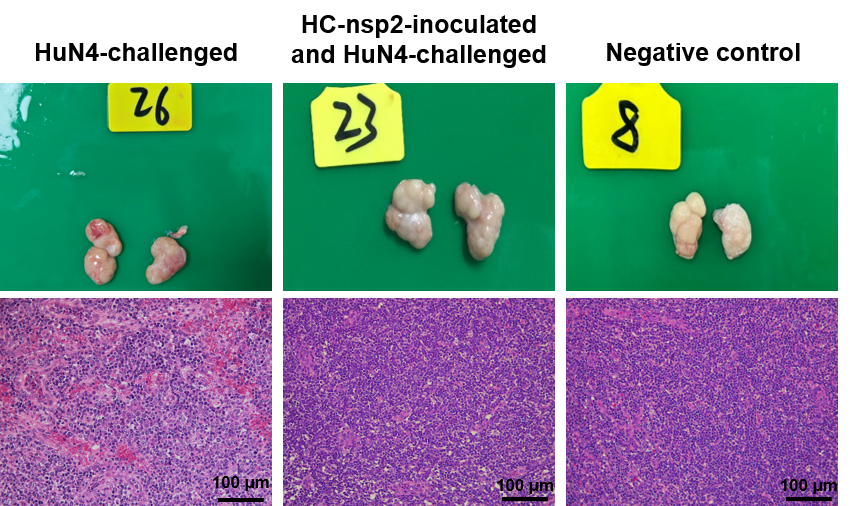


**Figure S5. The lymph nodes damage caused by the HuN4 challenge.** Representative gross submaxillary lymph nodes, and microscopic submaxillary lymph nodes lesions by H&E staining in each group were presented. Scale bars, 100 μm.


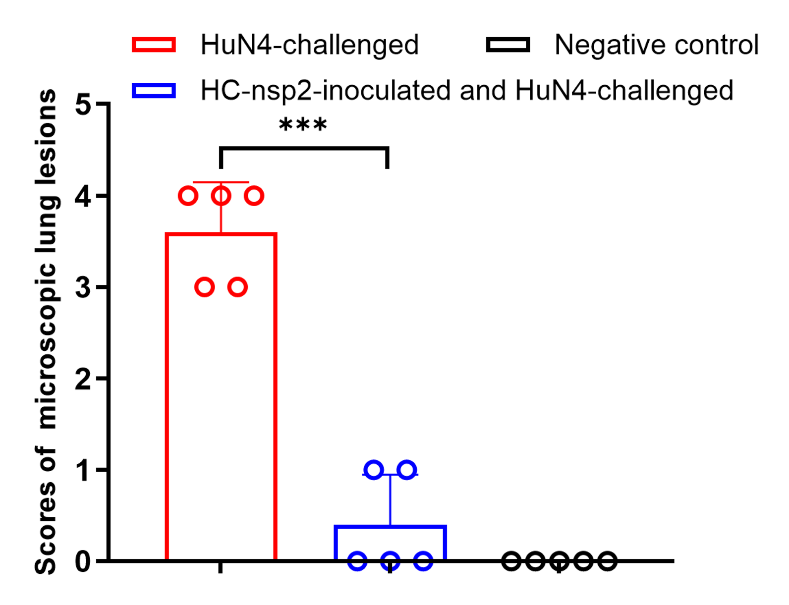


**Figure S6. The pulmonary lesion score of piglets in each group in animal challenge experiments.** The histopathological lung lesions of all piglets in each group were scored. An asterisk (*) indicates a significant difference between the groups (***: p < 0.001).


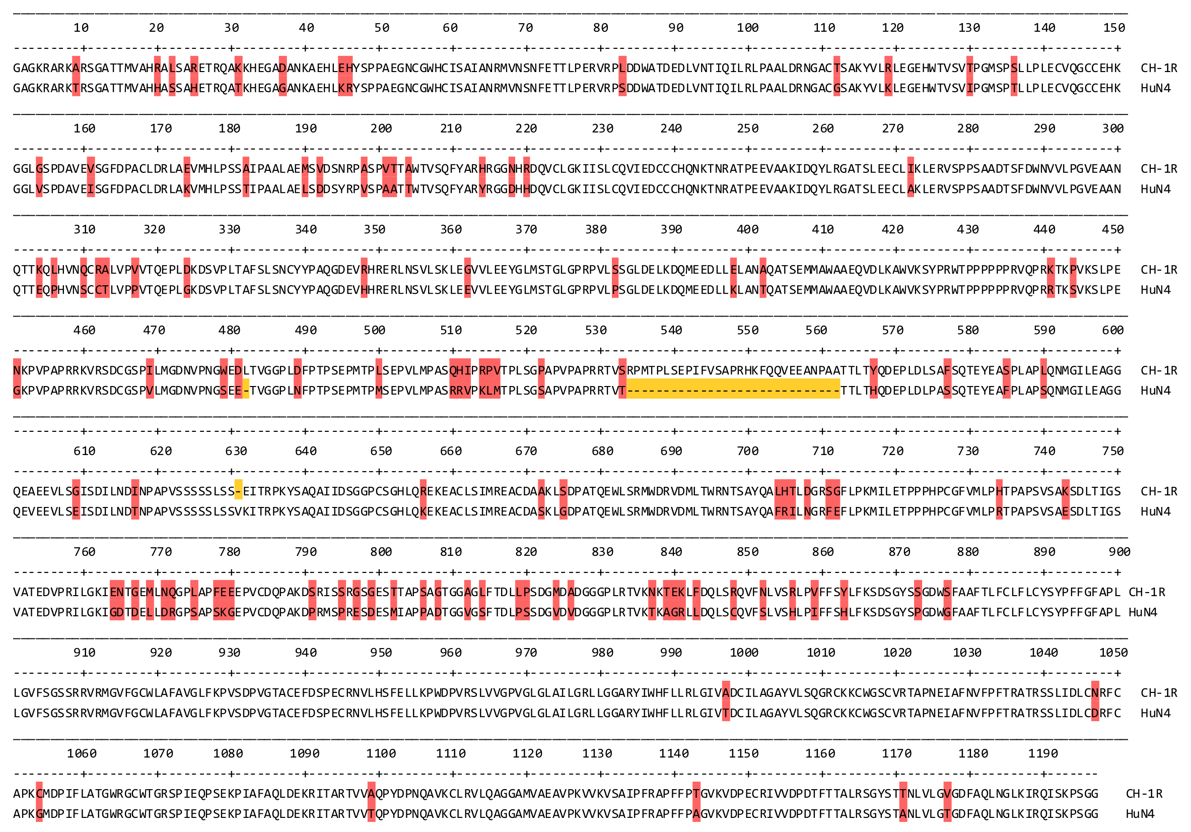


**Figure S7. Nsp2 amino acid alignment between HuN4 and CH-1R.** Red background indicates differences, and yellow background indicates deletions.

**Table S1. PCR primers used for infectious cDNA clone construction**

| Primers | Nucleotide sequence (5’-3’) |
| --- | --- |
| T-CH-F | CAACGGCAAGCAGCAAAAGA |
| T-CH-R | TTCAATTCAGGCCTAAAGTT |
| T-CH-(GP2-M)-F: | AACTTTAGGCCTGAATTGAAATGAAATGGGGTCTATGCAA |
| T-CH-(GP2-M)-R | TCTTTTGCTGCTTGCCGTTGTTATTTGGCATATTTAACAA |
| T-HC-F | CAACGGCAAGCAGCAAAAGA |
| T-HC-R | TTCAATTCAGGCCTAAAGTT |
| T-HC-(GP2-M)-F | AACTTTAGGCCTGAATTGAAATGAAATGGGGTCTATGCAA |
| T-HC-(GP2-M)-R | TCTTTTGCTGCTTGCCGTTGTTATTTGGCATATTTGACAA |
| T-CH1R-△nsp2-up-F | TAAAACGACGGCCAGTGCCA |
| T-CH1R-△nsp2-up-R | GCAATGAGGTGTGGGCCTCCACGCGTGTACCATTTGTGACTGCCAA |
| T-CH1R-△nsp2-down-F | TTGGCAGTCACAAATGGTACACGCGTGGAGGCCCACACCTCATTGC |
| T-CH1R-△nsp2-down-R | AGCTATGACCATGATTACGG |
| T-HuN4-△nsp2-up-F | TAAAACGACGGCCAGTGCCA |
| T-HuN4-△nsp2-up-R | GCAATGAGGTGCGGGCCTCCATTTAAATGTACCACTTATGACTGCCAA |
| T-HuN4-△nsp2-down-F | TTGGCAGTCATAAGTGGTACATTTAAATGGAGGCCCGCACCTCATTGC |
| T-HuN4-△nsp2-down-R | CAGCTATGACCATGATTACG |
| T-CH-nsp2-F | TTGGCAGTCACAAATGGTACGGTGCCGGAAAGAGAGCAAG |
| T-CH-nsp2-R | GCAATGAGGTGTGGGCCTCCCCCTGAAGGCTTGGAAATTT |
| T-HC-nsp2-F | TTGGCAGTCATAAGTGGTACGGTGCTGGAAAGAGAGCAAG |
| T-HC-nsp2-R | GCAATGAGGTGCGGGCCTCCTCCCGAAGGCTTGGAAATTT |

**Table S2. Group information for** **animal experiment of** **pathogenicity**

| Groups | Number of animals | Inoculate |
| --- | --- | --- |
| CH-1R-inoculated group | 5 | 2×10^5.0^ TCID_50_ per pig (CH-1R) |
| HuN4-inoculated group | 5 | 2×10^5.0^ TCID_50_ per pig (HuN4) |
| HC-nsp2-inoculated group | 5 | 2×10^5.0^ TCID_50_ per pig (HC-nsp2) |
| CH-nsp2-inoculated group | 5 | 2×10^5.0^ TCID_50_ per pig (CH-nsp2) |
| Negative control group | 3 | DMEM |

**Table S3. Group information for** **protective animal experiment**

| Groups | Number of animals | Inoculate | Challenge |
| --- | --- | --- | --- |
| HuN4-challenged group | 5 | DMEM | 2×10^5.0^ TCID_50_ per pig (HuN4) |
| HC-nsp2-inoculated and HuN4-challenged group | 5 | 2×10^5.0^ TCID_50_ per pig (HC-nsp2) | 2×10^5.0^ TCID_50_ per pig (HuN4) |
| Negative control group 2 | 3 | DMEM | DMEM |
